# Theoretical Prediction of Electronic Structure and Carrier Mobility in Single-walled MoS_2_ Nanotubes

**DOI:** 10.1038/srep04327

**Published:** 2014-03-10

**Authors:** Jin Xiao, Mengqiu Long, Xinmei Li, Hui Xu, Han Huang, Yongli Gao

**Affiliations:** 1 Institute of Super-microstructure and Ultrafast Process in Advanced Materials (ISUPAM), School of Physics and Electronics, Central South University, Changsha 410083, China; 2 Department of Physics and Astronomy, University of Rochester, Rochester, NY 14627, United States

## Abstract

We have investigated the electronic structure and carrier mobility of armchair and zigzag single-walled MoS_2_ nanotubes using density functional theory combined with Boltzmann transport method with relaxation time approximation. It is shown that armchair nanotubes are indirect bandgap semiconductors, while zigzag nanotubes are direct ones. The band gaps of single-walled MoS_2_ nanotubes are along with the augment of their diameters. For armchair nanotubes (5 ≤ *Na* ≤ 14), the hole mobility raise from 98.62 ~ 740.93 cm^2^V^−1^s^−1^ at room temperature, which is about six times of the electron mobility. For zigzag nanotubes (9 ≤ *Na* ≤ 15), the hole mobility is 56.61 ~ 91.32 cm^2^V^−1^s^−1^ at room temperature, which is about half of the electron mobility.

Since carbon nanotubes (CNTs) were discovered in 1991^1^, many nanomaterials, such as zero-dimensional nanoparticles, one-dimensional nanowires or nanotubes (NTs) as well as two-dimensional atomically-thick layers, have attracted intensive attentions due to their unique applications in microscopic physics and nanoscale devices^2^,^3^,^4^,^5^. Similar to graphene^2^, layered transition-metal dichalcogenides (TMDs), MX_2_ (M = Mo, W, Ta, or Nb, and X = S, Se, or Te) are considered as promising electronic and optoelectronic materials^5^,^6^,^7^,^8^,^9^,^10^,^11^ and have been synthesized^12^,^13^,^14^,^15^,^16^. MoS_2_, an important delegate of TMDs, arouses researchers' keen interest in recent years^15^,^16^,^17^,^18^,^19^. It is reported that monolayer MoS_2_ is a direct band gap semiconductor with a band gap value of 1.8 eV^20^,^21^. The experimental measurements on single-layer MoS_2_ transistors show a room-temperature electron mobility of at least 200 cm^2^V^−1^s^−1^, similar to that of graphene nanoribbons, and a room-temperature current on/off ratio up to 1 × 10^8^^9^. And the theory study find that electronic mobility is about 320 cm^2^V^−1^s^−1^ and the longitudinal acoustic phonon provides the largest scattering rates at room temperature in all electron-phonon coupling effect^22^. MoS_2_ nanotubes (NTs) are also semiconductors predicted by tight-banding (TB) method and density function theory (DFT), with more complex electronic structures and a band gap smaller than that of monolayer MoS_2_^23^,^24^. Due to their unique properties, some nano-materials and electronic devices based on TMD NTs excite scientists' interest^8^,^11^,^25^,^26^,^27^,^28^,^29^. However there are few reports on the mobility in TMD NTs, which is the central issue for such microelectronic semi-conducting materials.

In order to investigate the mobility in MoS_2_ NTs, we have carried out a study with the effect of phonon with first principles calculations using a density-function-based approach. In our research, we just consider about single-walled MoS_2_ NTs (in experiment, the typically MoS_2_ NTs are mutil-walled^14^) for the sake of study's convenience. Our results show that the hole mobility can even reach to ~740 cm^2^V^−1^s^−1^ and much higher than the electron mobility, which suggest that the MoS_2_ NTs could be considered as a good candidate of next electronic material.

## Results

**Figure 1:**
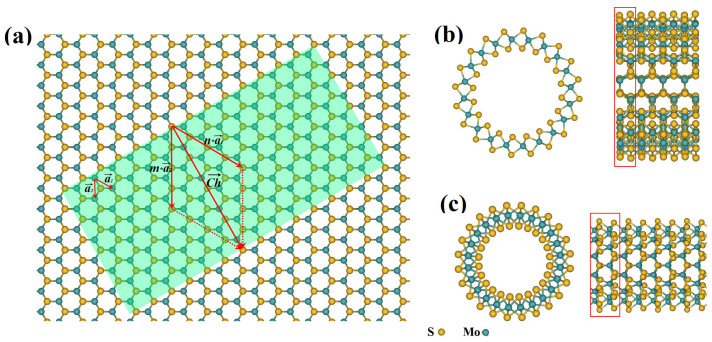
(a) The chiral vectors of MoS_2_ nanotubes (NTs), by which MoS_2_ NTs can be classified as (b) Armchair MoS_2_ NTs and (c) Zigzag MoS_2_ NTs. The rectangle is a cell of NTs.

Single-walled MoS_2_ NTs can be classified as chiral nanotubs, armchair nanotubes (ANTs) and zigzag nanotubes (ZNTs)^30^,^31^ based on their chiral vectors, as shown in Fig. 1. That is the same as CNTs. We use *Na* which is the coefficient of basic vectors in the chiral vector 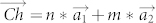 to signify the different size of ANTs (*n* = *m* = *Na*) and ZNTs (*n* = *Na*, *m* = 0). In our calculation, unit cells of NTs which we choose are shown in Fig. 1b and c. For an ANTs-*Na* unit cell, there are 2 × *Na* MoS_2_ units and they have the same lattice length (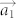), as shown in Fig. 1a. It is the same for ZNTs-*Na* except for the lattice length of 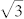 times of 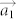.

**Figure 2:**
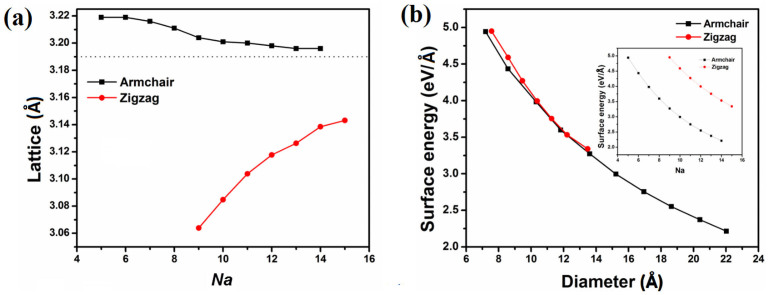
(a) The lattice length of MoS_2_ NTs as a function of *Na*. The lattice length of mono-layer MoS_2_ primitive cell is 3.19Å (the dot line) and that of bulk MoS_2_ is 3.16 Å^19^. In order to compare with each other, the lattice length of ZNTs is divided by 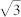. (b) Surface energy of MoS_2_ NTs as a function of diameter of tubes.

We investigate the geometry and energy of single-walled MoS_2_ NTs and find the lattice length evolution as a function of *Na* for both ANTs and ZNTs, shown in Fig. 2a. Compared with that of mono-layer MoS_2_ (3.19Å, the dot line in Fig. 2a) and bulk MoS_2_ (3.16Å)^19^, the lattice length of ANTs is getting longer, that means ANTs are stretched, while it is getting shorter for ZNTs, that means ZNTs are compressed. Both strains decrease with *Na* increasing. It is also supported by the surface energy results shown in Fig. 2b. The surface energy is defined as: 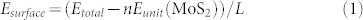
In which *E_total_* is the total free energy of nanotube cell, *E_unit_*(MoS_2_) is the free energy of primitive cell of mono-layer MoS_2_ crystal, *n* is the number of MoS_2_ units in a cell, *L* is the length of a cell. The surface energy can be considered as the energy of rolling a plane MoS_2_ sheet. The surface energy monotonically decreases with the diameter of NTs (Fig. 2b), regardless of their chiral. It indicates that the axial force of stretch or compression is getting smaller when the diameter of NTs increases. The numerical results of energy are the same as studied by Zibouche^24^ when the unit is unified.

**Figure 3:**
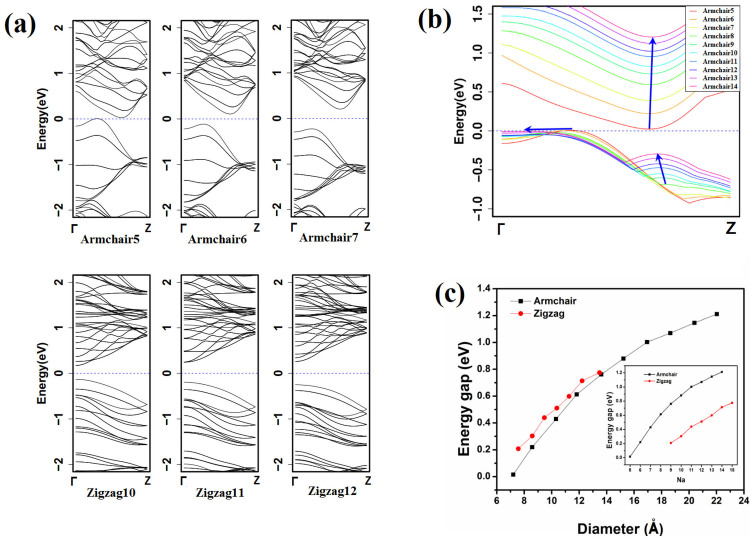
(a) Energy band structures of MoS_2_ NTs. (b) Energy bands near Fermi surface in MoS_2_ ANTs (5 ≤ *Na* ≤ 14). (c) The energy band gaps of MoS_2_ NTs. The energy gap of monolayer MoS_2_ and bulk MoS_2_ is 1.9 eV and 1.2 eV^32^, respectively.

The electronic structures of NTs are always associated with their chiral, like CNTs. The electronic structures of single-walled MoS_2_ NTs are shown in Fig. 3. The energy gap is open for both ANTs and ZNTs as shown in the energy band spectra (Fig. 3a). It can be found that MoS_2_ ANTs are indirect-gap semiconductors while ZNTs are direct-gap ones at Γ point. This result is consistent with previous studies by Seifert^23^ in TB and Zibouche^24^ in DFT. In our calculation, MoS_2_ ANT-5 whose energy gap is just 15.6 meV also can be considered as semiconductors because of bad gap underestimated by GGA. In 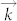 space (Γ to Z), there are two peaks in valence bands and one valley in conduction bands near the Fermi surface for ANTs (Fig. 3b). The energy band spectra of ANTs are similar as those of multi-layer MoS_2_^21^,^32^. The bottom of the valley, which is nearly at π/3 in the wave vector space, raises from 0 to 1.4 eV and shifts towards Z point slightly when *Na* increases from 5 to 14. In the valence bands, two peaks shift towards Γ point obviously, and the secondary peak near π/3 rises quickly with the increasing of *Na*. In two-dimension MoS_2_ multi-layer or mono-layer, the conduction band states at the K-point are mainly due to localized *d*-orbitals on the Mo atoms, relatively unaffected by interlayer coupling. However, the states near the Γ-point are due to combinations of the anti-bonding *p_z_*-orbitals on the S atoms and the *d* orbitals on Mo atoms, and have a strong interlayer coupling effect^33^. In MoS_2_ ANTs, there is the similar distribution of band states. The conduction band states are mainly due to localized on the Mo atoms and the states near the Γ-point are due to localized on the outer-side S atoms and Mo atoms. The states near at π/3 in wave vector space are due to localized on S atoms and Mo atoms, but it is asymmetric between outer and inner S atoms (Fig. S1 in Supporting Information (SI)). Because of the curve geometry, there are an equivalent vertical interlayer affection and an equivalent horizontal affection. The evolution of energy band spectra in ANTs can be considered as MoS_2_ transition from one-dimension tubes to two-dimension single layer. Due to the equivalent vertical interlayer affection, the energy evolution of band spectra is the same as that in MoS_2_ transition from three-dimension bulk to two-dimension single layer. The equivalent horizontal affection maybe is the reason of valleys (peaks) level shifting in wave vector space. However, the band spectra evolution in MoS_2_ ZNTs is very simple (Fig. S2 in SI). There is one valley or peak in the first Brillouin zone (BZ) for both of conduction bands (CB) and valence bands (VB). The expansibility of VB near Γ point is getting weaken with *Na* increasing. The energy gap monotonically increases with the diameter of NTs (Fig. 3c) and is insensitive with NT's chiral. The mono-layer MoS_2_ can be regarded as a nanotube with infinite diameter. Thus, the energy band spectra including the band gap of single-walled MoS_2_ NTs should approach to that of mono-layer MoS_2_^34^ when the diameter increases to infinity. But when the diameters are much large, the electronic states along the circumferential direction cannot be neglected^34^, and the energy band calculated based on two-dimensional reciprocal space sampling will be different with that of one-dimensional reciprocal space sampling. In our calculation, all of MoS_2_ nanotubes are considered as one-dimensional systems, and just K-points along the axial direction are calculated. So the band spectra of the armchair and zigzag MoS_2_ nanotubes will not tend to consistent with each other with the diameters of tubes increasing. Compared Fig. 3b with Fig. S2, this result can be deduced.

**Figure 4:**
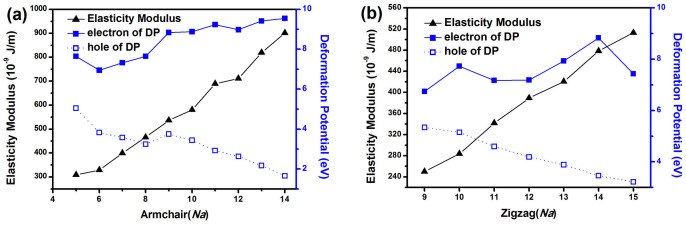
The elasticity modulus (*C*) and deformation potential (DP) of hole and electron for MoS_2_ ANTs (a) and ZNTs (b).

In our calculation, the stretching modulus *C* and deformation potential (DP) constant *E_1_* are necessary to calculate the relaxation time 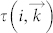 which is defined as *Formula* (3) in Methods section. In order to perform finite differentiation to obtain both *E_1_* and *C* (Fig. S3 in SI), additional total energies and band structures of the unit cell with the deformations of the lattice constant ±0.5% and ±1% are calculated. The *E_1_* and *C* of MoS_2_ ANTs and ZNTs are shown in Fig. 4a and 4b respectively. *C* is enhanced with diameter enlargement. Considering the cross-sectional area of NTs, the stretching modulus is about 144 ~ 234 GPa (see Table S1 in SI), which is in good agreement with the experiment results of 120 GPa by *Forro* *L*^35^. And theory result using density-functional-based tight-binding method by *Lorenz T*^36^ is that 209.7 GPa in ANTs-14 and 236.6 GPa in ZNTs-22. The differences with our result are acceptable because of the different energy calculation method and tube diameters (for ANTs-14, the diameter is 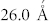 in *Lorenz T′* results, 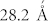 in ours). The DP constant of holes, which is about 5.1 ~ 1.6 eV and 5.3 ~ 3.2 eV in ANTs and ZNTs respectively, is smaller than that of electrons both for MoS_2_ ANTs (7.0 ~ 9.5 eV) and ZNTs (6.7 ~ 8.8 eV). And there is opposite tendency in DP change for electrons and holes.

**Figure 5:**
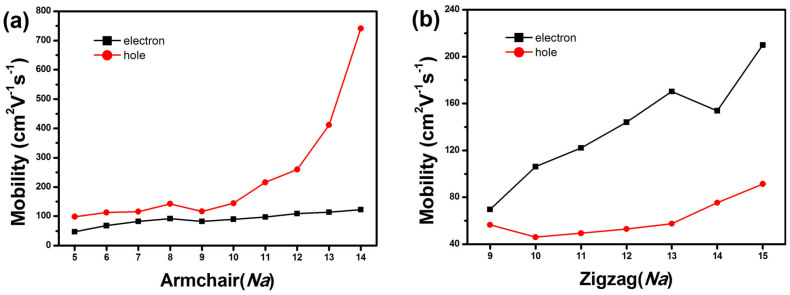
Charge mobility (*μ*) for holes and electrons in ANTs (a) and ZNTs (b) calculated by Boltzmann equation.

The carrier mobility (*μ*) of single-walled MoS_2_ NTs is predicted by Boltzmann transport equation (BTE) method without invoking the effective mass approximation. The results are outlined in Fig. 5. For ANTs (5 ≤ *Na* ≤ 14), the hole mobility at 300 K increases rapidly from 98.62 to 740.93 cm^2^V^−1^s^−1^ and is higher than electron mobility which increases from 46.50 to 121.77 cm^2^V^−1^s^−1^ (Fig. 5a). Whereas, for ZNTs (9 ≤ *Na* ≤ 15), the hole mobility is lower than electron mobility (Fig. 5b). Also the tendency of mobility with diameter is increscent for both polarities, in our theoretically prediction. Due to the stiffness enhance, there is longer relaxation time. And the stiffness is linearly relationship with diameter. So the carrier mobility increases with diameter enlargement. The mobility of MoS_2_ NTs is comparable with that of single-layer MoS_2_ which is detected to be at least 200 cm^2^V^−1^s^−1^ at room-temperature^9^. The room-temperature mobility of WS_2_ NTs is 760 cm^2^V^−1^s^−1^ in vacuum condition^11^. Our results are reasonable considering the same structures of MoS_2_ and WS_2_ NTs, as well as the opposite tendency of MoS_2_ NTs mobility for electrons and holes.

## Discussion

The carrier mobility is associated with band spectra, *C* and *E_1_*. For a given type of NTs, the difference of two carriers' mobility is mainly affected by band spectra. The relationship between mobility of NTs and *Na* is mainly affected by the ratio of *C* and *E_1_*. For a specific type of MoS_2_ tubes, the band spectra are similar but ratios of *C* and *E_1_* are monotonous increasing with *Na*. So the charge carrier mobility in both of MoS_2_ ANTs and ZNTs increases with *Na*. In MoS_2_ ANTs, the extensibility of VB is better than that of CB. The span of VB near Fermi surface is more than 1 eV, but that of CB is less than 1 eV. So the band structure is in favor of hole transport. The *E_1_* is also beneficial for hole. Two factors make hole carrier move faster in MoS_2_ ANTs. In MoS_2_ ZNTs, the span of VB near Fermi surface is about 0.6 eV, and that of CB is about 0.8 eV. Due to that the extensibility of CB is better than that of VB, the electron mobility is higher than hole mobility.

**Figure 6:**
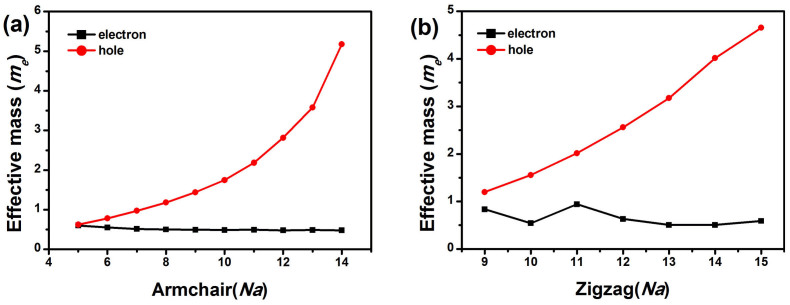
The effective mass (m*) of carriers in MoS_2_ ANTs (a) and ZNTs (b).

The mobility sometimes can be described very well by effective mass. Based on the band structures, we use parabolic fitting near the Fermi surface to calculate the effective mass of charge carriers (Fig. S4 in SI). The effective masses of electron and hole carriers are shown in Fig. 6. In our calculation, while the effective mass of electron carrier decreases slowly from 0.60 to 0.48 *m_e_* (*m_e_* is the mass of a free electron), the effective mass of hole carrier rapidly raises from 0.62 to 5.18 *m_e_* for ANTs with 5 ≤ *Na* ≤ 14 (Fig. 6a). Because of the bad parabolic nature at the top of valence band, the effective mass of hole may not be precise in MoS_2_ ANTs (Fig. S4 left in SI). For ZNTs with 9 ≤ *Na* ≤ 15, the effective mass of electron carrier is oscillatory from 0.51 to 0.94 *m_e_*, and the effective mass of hole carrier raises linearly from 1.20 to 4.66*m_e_* (Fig. 6b).

Based on the effective mass, we also calculate the mobility in Ref. SI (Fig. S5). The motilities predicted by effective mass are accord with that predicted by BTE method. In MoS_2_ ANTs, the mobility is about 56.04 ~ 145.88 cm^2^V^−1^s^−1^ for electron and 104.83 ~ 139.08 cm^2^V^−1^s^−1^ for hole. The tendency of electron mobility deduced from the effective mass is similar to that by the BTE method. For *Na* > 10, the hole mobility is equal to the electron mobility, that is very different from the results calculated with the BTE method. This may be caused by the imprecise effective mass of hole. In MoS_2_ ZNTs, the mobility is about 37.17 ~ 115.19 cm^2^V^−1^s^−1^ for electron and 23.51 ~ 35.43 cm^2^V^−1^s^−1^ for hole. The electron mobility is higher than the hole mobility in MoS_2_ ZNTs, the same as predicted by BTE method.

**Figure 7:**
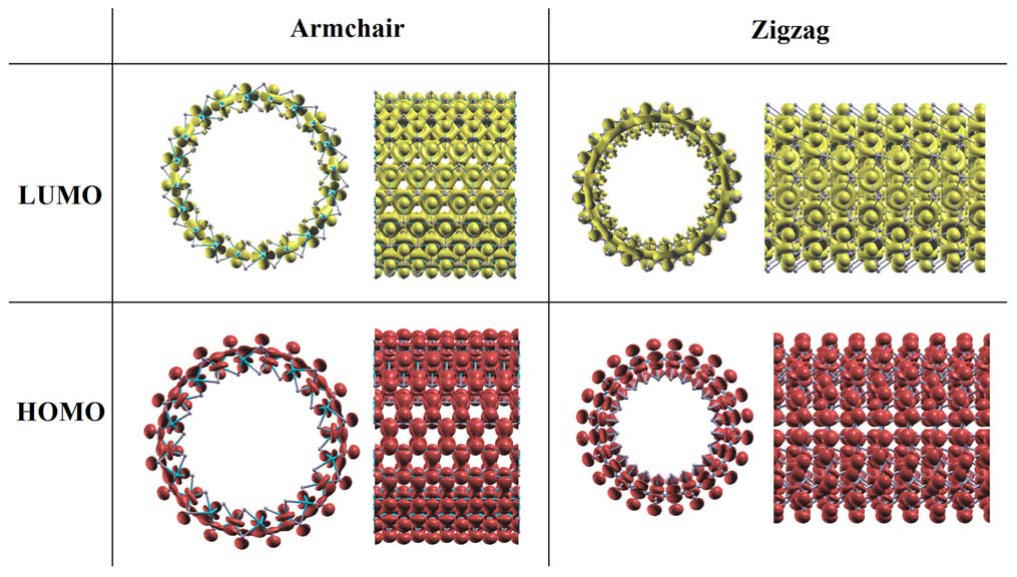
LUMO and HOMO of MoS_2_ ANTs-10 and ZNTs-12.

In order to understand the mobility tendency, the highest occupied molecular orbital (HOMO) and lowest unoccupied molecular orbital (LUMO) of single-walled MoS_2_ NTs are studied. Like a sandwich, there are three layers of atoms in MoS_2_ NTs. The outer and inner layers consisted of S atoms, signed as outer-S and inner-S, respectively; the middle layer is Mo atoms (middle-Mo). Analyzing HOMO and LUMO which are shown in Fig. 7 (we choose ANTs-10 and ZNTs-12 for examples), it is found that for both ANTs and ZNTs the HOMO is located on outer-S and middle-Mo layers. The difference is that while the HOMO of ANTs covers the bond of Mo-S (outer), that of ZNTs is isolated on Mo and outer side S atoms. It infers that ANTs are more beneficial for hole carriers' transport than ZNTs, so the hole mobility in ANTs is higher than that in ZNTs. The LUMO is located on middle-Mo layer for ANTs. Since the squeeze in inner-S layer, the major LUMO is located on middle-Mo layer and little is located on inner-S layer for ZNTs and cover the bond of Mo-S (inner side). Because of the better expansibility of HOMO than that of LUMO, the hole mobility in ANTs is higher than the electron mobility. Since the LUMO covers the bond of Mo-S in ZNTs, which is good for electron transport, the electron mobility is higher than the hole mobility. The LUMO of ANTs is more localized than that of ZNTs, so the electron mobility in ANTs is lower than that in ZNTs. Distributions of LUMO and HOMO mean that majority electron carriers transfer on the middle layer, and most of hole carriers transport on the outer surface of NTs. This kind of multi-layer material may be used to separate the electron and hole carriers.

In summarily, we have calculated the electronic structure and the intrinsic charge carrier mobility of single-walled MoS_2_ NTs with the effect of the longitudinal acoustic phonon, using first-principles density functional theory and the BTE with the relaxation time approximation. The numerical results indicate that the hole mobility can reach 740.93 cm^2^V^−1^s^−1^ at room temperature for MoS_2_ ANTs-14 which is almost an order of magnitude higher than the electron mobility. But for MoS_2_ ZNTs, the hole mobility at room temperature is just half of the electron mobility. We also find that the charge mobility increases with the diameter in MoS_2_ NTs. Because of the huge difference mobility in hole and electron, MoS_2_ ANTs can be considered as *p*-type semiconductors and MoS_2_ ZNTs can be considered as *n*-type semiconductors. Due to the high carrier mobility, MoS_2_ NTs maybe are the advanced material to design electronic element.

## Methods

Optimized geometries and band structures are implemented by the Vienna *ab-initio* simulation package (VASP)^37^. The generalized gradient approximation (GGA)^38^ with the Perdew-Wang (PW91)^39^ exchange correlation function is chosen. And some important parameters are tested in Fig. S6. The criterion of convergence is that the residual forces are less than 0.01 eV/Å and the change of total energy is less than 10^−4^ eV. The vacuum space between two adjacent NTs is set at least 10Å to eliminate the interactive effect on each other. The lattice length of single-walled MoS_2_ NTs is optimized based on searching the lowest total energy, as shown in Fig. S3(a). We also compare some physics quantities calculated by LDA with GGA, the results shown in Table S2 and the correlative discussion shown in *SI*. We find the consistent conclusion should be got using both of LDA and GGA.

The charge transport has been dealt with by a band-like model, in which the electron–phonon coupling is regarded as a perturbation and the charge is delocalized over the crystal. Assuming the thermal electron wavelength is close to the acoustic phonon's wavelength, we consider only transport at room temperature and focus on the electron–acoustic phonon coupling in the framework of DP^40^, where three major approximations have been assumed: (i) the transverse acoustic (TA) phonon mode is not included due to its negligible effect on the DP; (ii) the scattering probability is independent of state momentum; (iii) charge transport direction is assumed to be parallel to the phonon propagation direction^41^,^42^. The carrier mobility is calculated by BTE method beyond the effective mass approximation which is used to predict the mobility of semiconductor nanometerials, like graphene, CNTs and so on^42^,^43^,^44^,^45^,^46^,^47^. Within the BTE method, the carrier mobility *μ* in the relaxation time approximation can be express as [Ref. 46 and 48]: 
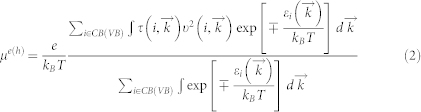
Where the minus (plus) sign is for electron (hole). 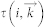 is the relaxation time, 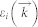 and 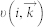 are band energy and the component of group velocity at 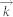 state of the *i*th band, respectively. The summation of band was carried out over VB for hole and CB for electron. Furthermore, the integral of 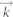 states is over the first BZ. In order to obtain the mobility, three key quantities (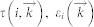 and 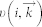) must be determined. In this work, the band energy 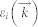 is calculated by density functional theory. The 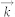-mesh is chosen as 1 × 1 × 200, which is fine enough to give converged relaxation time and mobility. The group velocity of electron and hole carriers can be obtained from the gradient of the band energy 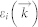
in 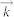-space, 
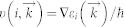
. The relaxation time 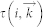 is calculated by the collision term in the Boltzmann method, and within the DP formalism, it can be expressed as^42^,^46^: 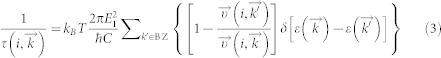
Here the delta function denotes that the scattering process is elastic and occurs between the band states with the same band index. *E*_1_ is the DP constant of the *i*-th band, and *C* is the elastic constant. In principle, different scattering channels can be added in the following way: 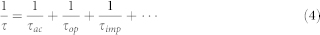
where ac, op, and imp denote acoustic, optical phonons, and impurity respectively. It is well known that the mobility is major effect by the smallest relaxation time (τ). In the room temperature, the corresponding electron wavelength is close to the acoustic phonon wavelength and much larger than the lattice constant. So we can deal with the effects of the longitudinal acoustic phonon as the uniform deformation of lattice approximatively, and the band model combined with DP theory is valid at the room temperature. Also in monolayer MoS_2_ research, Xiaodong Li *et al.*[Ref 22] has reported the lowest optical phonon energy is about 35 meV, and the longitudinal acoustic phonon provides the largest scattering rates at room temperature. So, we only consider the acoustic phonon scattering in our calculation.

## Author Contributions

J.X. carried out the first-principles calculations, prepared all figures and wrote the manuscript. M.L. revised the manuscript and directed this work. X.L., H.X., H.H. and Y.G. involved in discussion. All authors analyzed the results and reviewed the manuscript.

## Supplementary Material

Supplementary Information
