# Supplementary material for: Theoretical Prediction of Electronic Structure and Carrier Mobility in Single-walled MoS2 Nanotubes
Source: Sci Rep. 2014 Mar 10;4:4327. doi: 10.1038/srep04327 (PMC3948089; doi:10.1038/srep04327)
Supplement: Supplementary Information [file srep04327-s1.docx]

# Support Information

Theoretical Prediction of Electronic Structure and Carrier Mobility in Single-walled MoS_2_ Nanotubes

*Jin Xiao*^1^*, Meng-Qiu Long*^1,^a^)^*, Xin-Mei Li*^1^*, Hui Xu*^1,^b*^)^, Han Huang*^1^ *and Yong-Li Gao*^1,2^

^1^Institute of Super-microstructure and Ultrafast Process in Advanced Materials (ISUPAM), School of Physics and Electronics, Central South University, Changsha 410083, China

^2^Department of Physics and Astronomy, University of Rochester, Rochester, NY 14627, United States

Corresponding author: ^a)^[mqlong@csu.edu.cn](mailto:mqlong@csu.edu.cn) b^)^ [cmpxhg@csu.edu.cn](mailto:cmpxhg@csu.edu.cn)


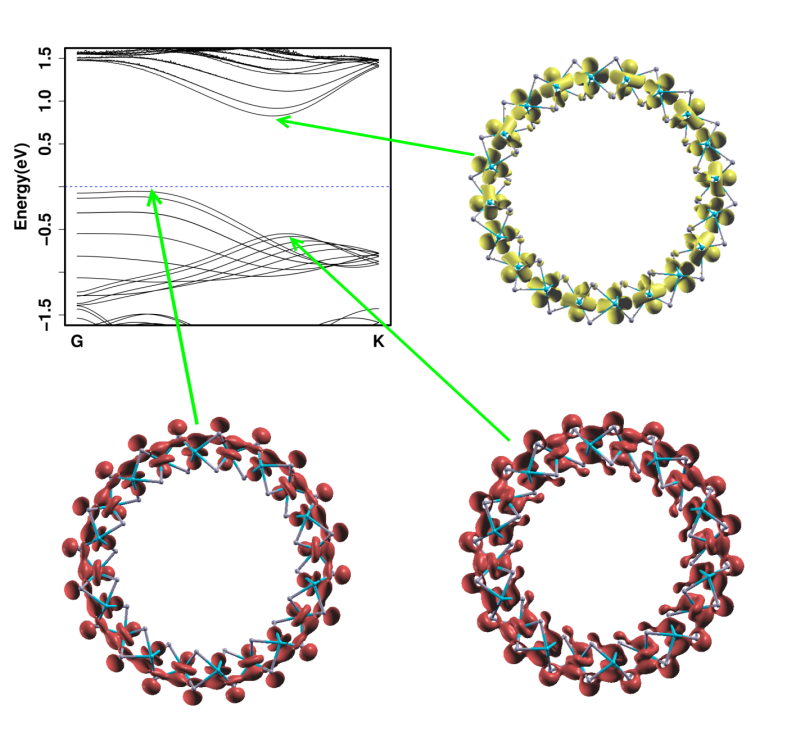


**Fig S1.** The band states near valleys and peaks of band spectra in MoS_2_ ANTs-10.


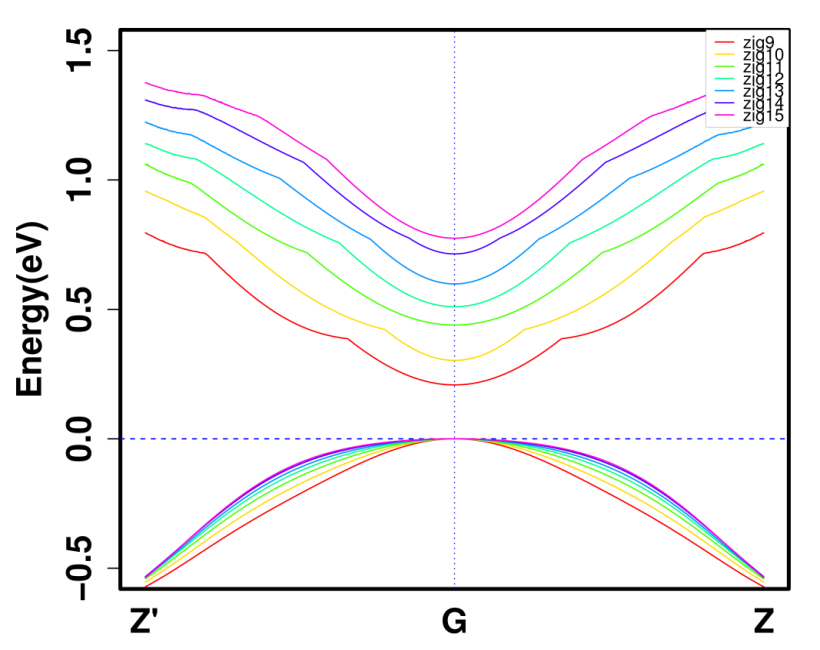


**Fig S2.** Energy bands near Fermi surface in MoS2 ZNTs (9≤Na≤15).


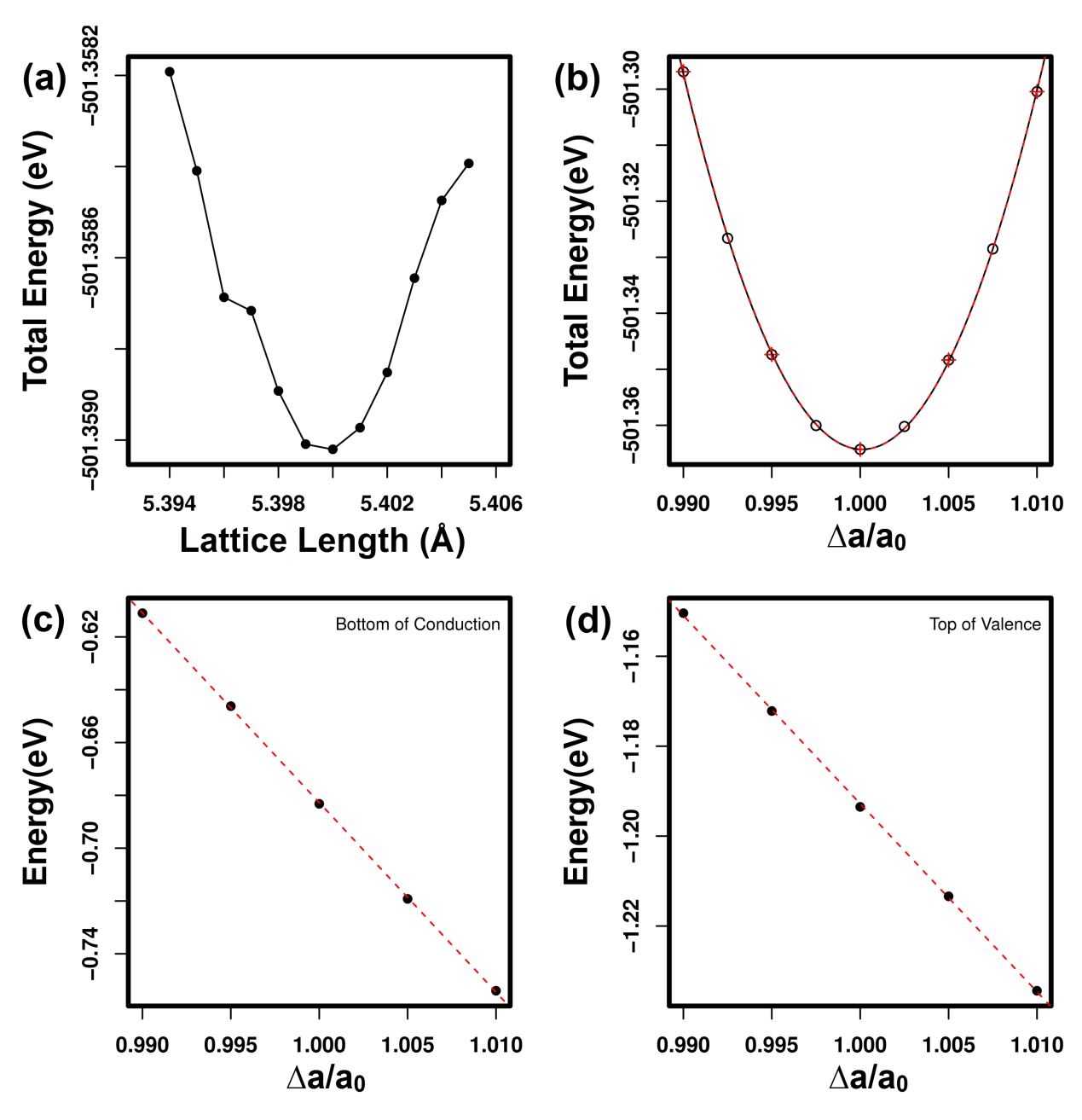


**Fig S3.** The best lattice length, elastic constant fitting and DP constant for MoS_2_ ZNTs-12. (a) The total energy as a function of lattice length to find the best lattice length. (b) The total energy of unit cell as a function of lattice deformation along axis direction. The red dash line and black solid line is parabola fitting with five points (red +) and nine points (black ○) respectively. Based on the parabola fitting, the elastic constant can be obtained. (c) The band edge position of conductive bottom with respect to the lattice dilation ∆a/a_0_. (d) The band edge position of valence top with respect to the lattice dilation ∆a/a_0_. The red dash line is linear fitting. Based on linear fitting, the DP constant of electron and hole carries can be obtained.


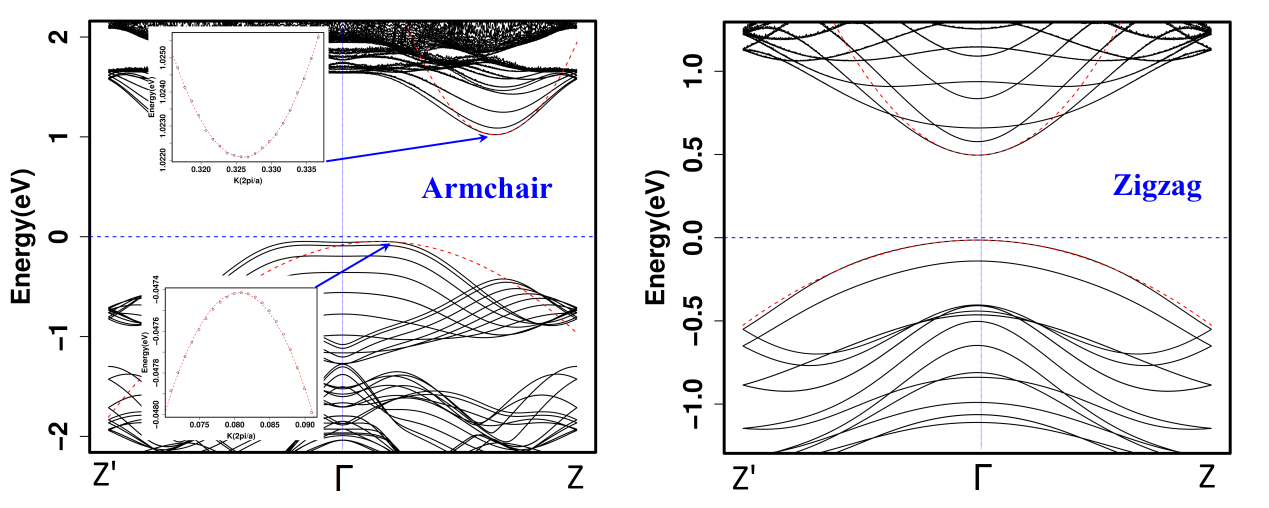


**Fig S4.** The parabolic fitting at the top of valence band and bottom of conduction band for MoS_2_ NTs. The red dash lines are the parabolic fitting lines. The MoS_2_ ANTs-10 and ZNTs-12 are shown as examples.


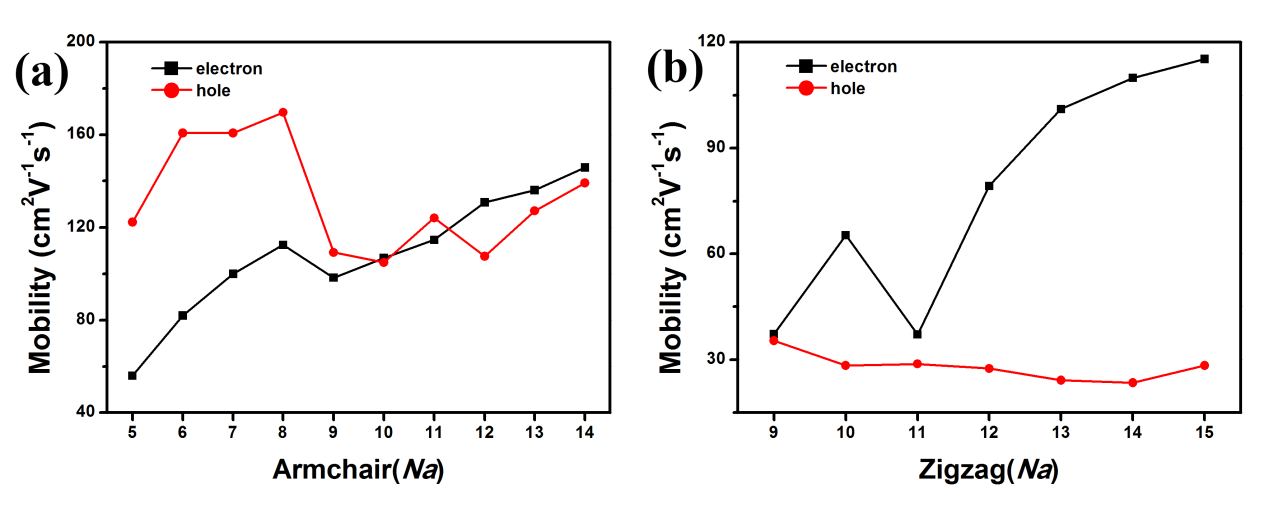


**Fig S5.** The mobility for holes and electron in MoS_2_ ANTs and ZNTs calculated by effective mass method.


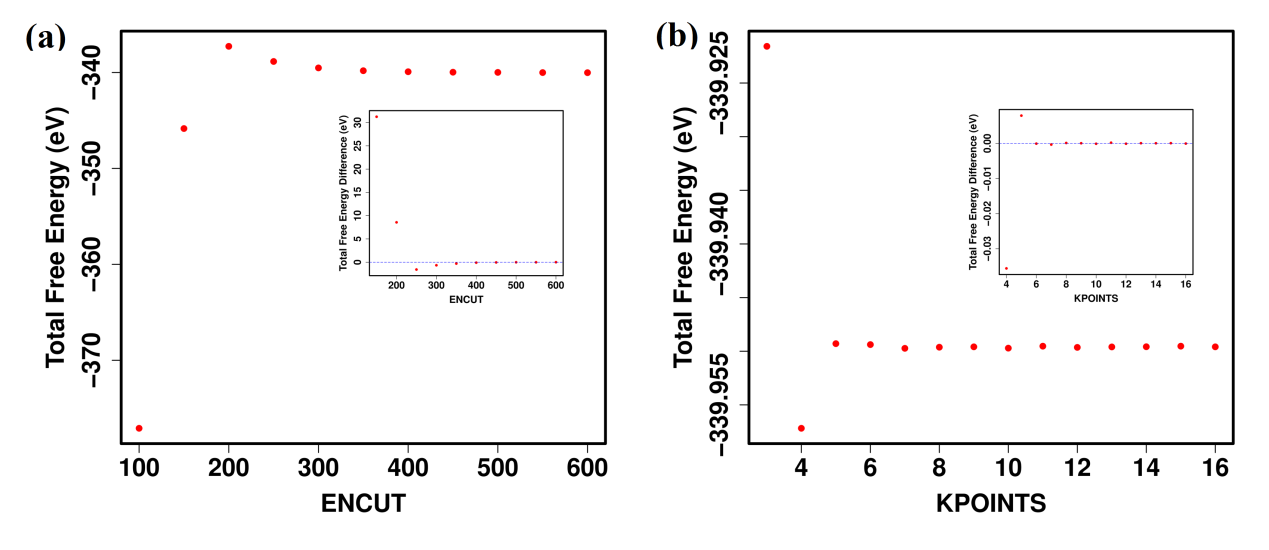


**Fig S6.** The total energy as a function of energy cutoff (ENCUT) (a) and KPOINTS (b), the insert pictures are the differential energy.

**Table S1**. The Elasticity Modulus (*C*) of MoS2 nanotubes.

|  | Diameter(Ǻ) | *C* (GPa) |
| --- | --- | --- |
| ANTs-5 | 12.96 | 234.22 |
| ANTs-6 | 14.52 | 198.64 |
| ANTs-7 | 16.28 | 192.08 |
| ANTs-8 | 17.88 | 185.51 |
| ANTs-9 | 19.68 | 176.19 |
| ANTs-10 | 21.34 | 162.13 |
| ANTs-11 | 23.07 | 164.89 |
| ANTs-12 | 24.79 | 147.27 |
| ANTs-13 | 26.56 | 147.89 |
| ANTs-14 | 28.21 | 144.25 |
| ZNTs-9 | 13.36 | 178.34 |
| ZNTs-10 | 14.51 | 171.51 |
| ZNTs-11 | 15.44 | 182.50 |
| ZNTs-12 | 16.39 | 184.48 |
| ZNTs-13 | 17.25 | 179.88 |
| ZNTs-14 | 18.29 | 181.95 |
| ZNTs-15 | 19.49 | 171.96 |

**Table S2** The results of zigzag-12 MoS_2_ nanotube with GGA and LDA. L is the lattice length. C is Elastic modulus. DP^(h)^ is deformation potential of hole. DP^(e)^ is deformation potential of electron. ∆ is the energy gap. µ^(e)^ is the mobility of electron carriers at 300K. µ^(h)^ is the mobility of hole carriers at 300K.

|  | L(Å) | C(10^-9^ J/m) | DP^(e)^ (eV) | DP^(h)^ (eV) | ∆ (eV) | µ^(e)^ (cm^2^V^-1^s^-1^) | µ^(h)^ (cm^2^V^-1^s^-1^) |
| --- | --- | --- | --- | --- | --- | --- | --- |
| GGA | 5.400 | 389.328 | 4.18496 | 7.18208 | 0.510275 | 143.851 | 52.9385 |
| LDA | 5.276 | 409.200 | 4.28496 | 7.26128 | 0.597721 | 152.653 | 38.8296 |

**Calculation parameters:**

We test the parameters of VASP for the MoS_2_ system, shown in Fig. S6. We find when ENCUT≥400, the total energy is converged. So in our calculation, ENCUT=400 is chosen. When KPOINTS≥5, the total energy is almost unchanged. In our calculations, we choose 1×1×5 for structure optimization, 1×1×11 for electronic self-consistent calculation and 1×1×200 for energy band calculation.

We calculate the total energy of MoS_2_ NTs with different lattice length. We choose MoS_2_ ZNTs-12 as an example, shown in Fig. S3. All of MoS_2_ NTs with specified lattice length are optimized. Then based on the total energies of optimized MoS_2_ NTs, we choose the best lattice length, with which the total energy is the lowest one, for our following calculation, as shown in Fig. S3(a). We calculate the total energy and band structure of unit cell with deformations of the lattice constant ±0.25%, ±0.5%, ±0.75% and ±1%. Based on $C=a_{0}\left( {\partial^{2}E}/{\partial a^{2}} \right)|_{a=a_{0}},$we use parabola fitting of total energy to obtain the elastic constant, as shown in Fig. S3(b). In Fig. S3(b), the fitting lines with five points (red dashing line) and nine points (black solid line) overlap with each other. So in order to save resources, we choose five points (±0.5%, ±1% and 0) in our following calculation. We use linear fitting of valence and conductive bands to get DP constant, as shown in Fig. S3(c, d).

**Effective mass fitting:**

We choose 21 points which the interval is 0.001×2π/a_0_ to fit bands near the Fermi surface. Based on $m^{*}=\hbar\left[ {\partial^{2}E(k)}/{\partial k^{2}} \right]^{-1},$ the effective mass can be obtained from parabolic fitting, as shown in Fig S4.

**Calculation of carrier mobility based on effective mass:**

With the deformation potential (DP) approach and effective mass approximation, the charge carrier mobility *µ* of one-dimension crystal can be express as

$\mu=\frac{e\tau}{m^{*}}=\frac{e\hbar^{2}C}{{(2\pi k_{B}T)}^{1/2}\left| m^{*} \right|^{3/2}E_{1}^{2}}$ (S1)

in which *τ* is the acoustic phonon scattering relaxation time and $m^{*}=\hbar\left[ {\partial^{2}E(k)}/{\partial k^{2}} \right]^{-1}$ is the effective mass of charge. $C=a_{0}\left( {\partial^{2}E}/{\partial a^{2}} \right)|_{a=a_{0}}$ is the stretching modulus of one-dimension crystal and $a_{0}$ is the lattice constant. The DP constant *E_1_* is obtained from $\delta E(k_{F})=E_{1}({\delta a}/{a_{0}})$, *E*(*k_F_*) is the energy band near the Fermi surface. The method to obtain *C* and *E_1_* is shown in Fig. S3. We also calculate the mobility in MoS_2_ NTs using formula (S1), as shown in Fig. S5. The motilities predicted by two different methods are in the same order of magnitudes.

**Compared LDA with GGA**

We test zigzag-12 MoS_2_ nanotubes with LDA and GGA. The results are shown in Table S2. In this case, the lattice length with LDA is 5.276Å which is small 2.3% than GGA result. The modulus calculated by LDA is larger 5.1% than that of GGA due to the small lattice length. The energy gap of LDA is 0.597721 eV which is according with the truth: the energy gap with LDA potential is overrated and the energy gap with GGA potential is underestimated. The deformation potentials of two particles with LDA are few differences with those calculated by GGA. The electron mobility with LDA is 152.653 cm^2^V^-1^s^-1^ which is a little higher than GGA result (143.851 cm^2^V^-1^s^-1^). That is because of the high modulus with LDA. The hole mobility with LDA is 38.8296 cm^2^V^-1^s^-1^ which is lower than that with GGA (52.9385 cm^2^V^-1^s^-1^). The main reason is the low precise to describe the conductive bands which are unoccupied orbits. With different exchange correlation potentials, the differences of physics quantities are acceptable. And the electron mobility is much higher than hole mobility using LDA. This relationship of two kind carriers’ mobility is the same as using GGA. So the consistent conclusion should be got using both of LDA and GGA.
